# Patterns of Living Alone in South Korea Compared to Other Countries: A Public Health Perspective and YouTube Topic Modeling Analysis

**DOI:** 10.3389/ijph.2025.1608509

**Published:** 2025-08-29

**Authors:** Heesook Son, Sangmin Song, YoungBin Kim, Joohyun Chung

**Affiliations:** ^1^ Red Cross College of Nursing, Chung-Ang University, Dongjak-gu, Republic of Korea; ^2^ Graduate School of Advanced Imaging Sciences, Multimedia and Film, Chung-Ang University, Dongjak-gu, Republic of Korea; ^3^ Elaine Marieb College of Nursing, University of Massachusetts Amherst, Amherst, MA, United States

**Keywords:** topic modeling, cultural differences, middle-aged adults, single-person households, YouTube analysis

## Abstract

**Objectives:**

This study explored the experiences, challenges, and social dynamics of middle-aged individuals living alone in South Korea, compared with English-speaking individuals. From a public health perspective, this study examined cultural and generational factors influencing wellbeing.

**Methods:**

This cross-sectional descriptive study used topic modeling with natural language processing to analyze YouTube video comments related to single-person households collected from 30 Korean and 88 English-language channels.

**Results:**

Distinct cultural and generational differences emerged. Korean participants, particularly women, frequently expressed concerns about social stigma surrounding marriage and singlehood. English-speaking participants emphasized careers and personal independence. Themes included employment, financial security, travel, and food. Younger Koreans showed interest in leisure activities (mukbang, solo drinking). Older Koreans expressed concerns about economic stability and societal expectations.

**Conclusion:**

Understanding the cultural and generational context of living alone is essential for developing public health strategies addressing social isolation, mental health, and health equity. Digital platforms function as alternative social spaces, offering insights into socially isolated individuals’ needs. Public health interventions should consider online engagement to support wellbeing and reduce health disparities in this population.

## Introduction

The growing prevalence of single-person households has become a significant demographic trend worldwide, with notable increases across both Eastern and Western societies. In South Korea, this trend is increasingly evident among adults aged 40 to 64, commonly defined as middle-aged [1], who have begun to live alone in greater numbers. A middle-aged single-person household refers to an individual in this age group who lives alone without cohabiting with others. According to Statistics Korea, the national proportion of such households has gradually increased from 31.2% in 2020 to 35.5% in 2023 [2–5]. Specifically for middle-aged adults (aged 40 to under 65), the proportion increased from 10.2% in 2010 to 16.1% in 2020, then to 22.1% in 2022, and most recently reached 25.5% in 2024 [5]. Similarly, in 2022, there were 37.9 million single-person households in the United States, accounting for 28.9% of all households, compared to just 13.6% in 1962 [6]. The percentage of 45-year-olds living alone has increased for both women and men since 1980. Among women, the proportion rose from 5.2% in 1980 to 9% in 2000, before slightly declining to 7.6% in 2018. For men, the proportion increased from 7.1% in 1980 to 12.1% in 2000, then decreased slightly to 10.7% in 2018. Despite the small declines after 2000, the overall trend indicates a significant rise in solo living among middle-aged adults, particularly among men [7, 8]. Changing family structures, societal expectations, and lifestyle patterns have contributed to a growing number of middle-aged adults living alone. While much attention has been given to older adults, who do have high rates of living alone, recent trends show a significant rise in single-person households among those in middle age as well. For instance, life expectancy differences by sex play a role in long-term living arrangements [9], especially later in life; in 2023, women in the U.S. had an average life expectancy of 80.2 years, compared to 74.8 years for men, and in Korea, 86.4 years for women versus 80.6 for men [10, 11]. However, the increase in solo living is no longer limited to older populations, and these shifts are becoming more visible among middle-aged individuals as well.

Living alone presents distinct health challenges, especially for middle-aged and older adults, manifesting as lower physical activity levels, increased mental health risks, and diminished social support compared with those living in multi-person households [12–15]. Previous studies have reported higher rates of depressive symptoms and suicidal ideation among individuals in single-person households, highlighting the urgent need for targeted interventions to support wellbeing in this population [13, 14]. Additionally, individuals living alone experience a lower quality of life than those in multi-person households [15]. Despite these risks, much of the public health focus has been on older adults living alone [16–21], as they are often included in institutional support systems through healthcare and welfare programs [18, 22]. However, middle-aged individuals living alone frequently fall outside these support structures and remain overlooked, despite their heightened vulnerability to future health risks, particularly those with poor health and insufficient retirement preparation [23].

Cultural factors also shape experiences of living alone, as they influence societal perceptions and personal wellbeing [24, 25]. In South Korea, where collectivist cultural values and intergenerational co-residency remain deeply embedded, marriage continues to function as a key marker of adulthood and social legitimacy [26]. Within this cultural framework, individuals, particularly women who remain unmarried beyond the socially expected age, may be perceived as emotionally unfulfilled, socially adrift, or lacking the familial bonds considered essential to normative life trajectories [27, 28]. While growing numbers of women have begun to frame solo living as a form of independence and personal choice, such narratives remain fragile in the face of enduring gendered expectations. In particular, never-married or divorced women, unlike their widowed counterparts, are frequently viewed as having failed to meet socially prescribed milestones, leading to subtle forms of exclusion from family networks, community life, and broader systems of social recognition [27]. These cultural dynamics help explain why the relationship between marriage and happiness varies significantly across countries, likely reflecting differences in societal norms and attitudes toward living alone [29]. However, the intersection of sex and solo living among middle-aged adults remains relatively underexplored, with few studies in the U.S. or other countries addressing this specific demographic. One from the BBC is still reflected in newspaper articles like ‘Single shaming: Why people jump to judge the un-partnered’ [30]. In addition, alternative family structures and living arrangements, such as “living-apart-together” relationships and the increasing acceptance of the LGBTQIA+ community, have become more prevalent in recent years [31–33].

In more individualistic cultures like the United States, living alone is generally viewed as a symbol of autonomy and self-reliance, with fewer societal expectations to conform to traditional family roles [34]. These differing cultural attitudes affect not only the decisions individuals make regarding living arrangements but also how society perceives and supports them. For example, the growing acceptance of solitary dining in South Korea through trends such as mukbang reflects changing social attitudes and attempts to normalize solo living experiences [34, 35].

Despite the global rise in single-person households, little research has focused on middle-aged individuals living alone, particularly regarding the cultural contexts that influence their daily lives [28]. Although previous studies have primarily used surveys or cross-sectional analyses to examine this population, deeper insights into their lived experiences, interests, and challenges are needed. Understanding these factors is essential for developing tailored interventions that can support the quality of life and wellbeing of this group, especially in terms of financial security, caregiving needs, and social participation [15]. From a public health perspective, such insights are critical to reducing health disparities and promoting long-term health equity.

Social media platforms provide valuable opportunities to explore the everyday experiences of individuals living alone. In particular, YouTube offers diverse content, such as daily life vlogs and interactive comments, which reflect the interests and concerns of single-person households. Given the increasing role of digital spaces in facilitating social interactions, especially among socially isolated populations, analyzing YouTube content can provide meaningful public health insights into how people manage their lives alone. Thus, this study aimed to examine the life interests and concerns of middle-aged individuals living alone in South Korea and English-speaking countries by analyzing YouTube video comments. By identifying cultural and generational patterns in these discussions, this research can contribute to improving the understanding of the public health implications of living alone and supporting the development of culturally responsive strategies to promote wellbeing in this growing population.

## Methods

### Study Design

This cross-sectional descriptive study utilized topic modeling with natural language processing to analyze YouTube video comments related to single-person households among middle-aged individuals in South Korea and compared those with comments from English-speaking individuals in other countries. Middle-aged individuals were defined as those between 40 and 65 years old [1]. To identify relevant content, we conducted a keyword-based search on YouTube using terms such as (“middle-aged,” “midlife” “middle adult” or “midlife”) and (“one-person residence,” “living alone,” “single-person household” “solo living” or “individually housed,” in both English and Korean. We included videos published between July 2023 and August 2023. Exclusion criteria were [1] videos focusing exclusively on young adults or older adults outside the defined middle-aged range, and [2] promotional or commercial content, such as advertisements. The final sample included 2,736 videos with 780,000 associated comments from Korea and 11,059 videos with 700,953 comments from English-speaking countries. The majority of comments originated from the United States, Canada, the United Kingdom, Australia, and New Zealand. Comments related to single-person households were identified using BERT-based topic modeling, a computational technique that groups semantically similar content by leveraging natural language processing. This method allowed us to extract dominant themes across a large volume of data.

### Data Sources

YouTube has been used globally for several years, gaining widespread popularity following its acquisition by Google in 2010. According to globalmediainsight.com, YouTube has facilitated the popularity of various content creators, including bloggers who endorse products to their audiences [36]. With over 2.7 billion monthly active users, this platform has grown substantially over time. Nearly 43% of global Internet users access YouTube every month [36].

We collected the titles and descriptions, excluding the video content itself, and user comments from YouTube videos published between July and August 2023. These videos and comments focused on the health-related social dynamics experienced by middle-aged adults living in single-person households. We selected 30 channels with a specific focus on middle-aged individuals living in single-person households in Korea. We also selected the 88 most popular YouTube channels with English-language content based on their number of subscribers. Our selection criteria were based on keywords. Regarding the demographic groups examined in this study, gender and age data were obtained through the YouTube Data API, which provides limited demographic information to researchers as part of the YouTube Researcher Program. Access to this data is strictly regulated and subject to YouTube’s privacy policies. All demographic insights used in this study were derived in full compliance with these guidelines.

The inconsistency in sampling criteria between the Korean and English-language videos largely reflects practical and contextual differences. Korean content explicitly featuring middle-aged individuals living alone was more readily available and clearly labeled, making targeted selection feasible. In contrast, English-language content on similar themes was less consistently categorized by age or living arrangement, prompting reliance on popularity metrics such as views and engagement to identify relevant material. The final sample included 2,736 videos with 780,000 associated comments from Korea and 11,059 videos with 700,953 comments from English-speaking countries. All data were compiled into a comma-separated values (CSV) file.

### Pre-Processing

We removed special characters (e.g., _, [, ], _, \), symbols, and numeric values that may not be relevant for analysis or could introduce noise into the data. Irrelevant links such as advertisements were eliminated, and all text was converted to lowercase to maintain consistency and simplify text analysis complexities.

### Data Feature Extraction

The numerically represented preprocessed text was used for the analysis. Log sampling was employed to ensure a more balanced representation of comments across YouTube channels. This big data technique selects a proportion of data based on logarithmic scaling, allowing for more equitable sampling across sources with varying levels of activity. The total number of comments per channel was first calculated, after which a logarithmic function was applied to determine the percentage of comments to retain from each channel. This method reduces the overrepresentation of channels with exceptionally high comment volumes while preserving meaningful patterns in the data. It also helps minimize sampling bias and supports the inclusion of a diverse range of content sources in the dataset. For example, if Video A had 10,000 comments and Video B had 100 comments, a linear sampling method would heavily favor Video A. However, with log sampling, Video A would be weighted as log (10,000) −4 and Video B as log (100) −2, narrowing the disparity in sampling weight. This method helped ensure that videos with moderate engagement were not overshadowed by viral content and allowed for more diverse perspectives in the comment dataset. Before proceeding to topic modeling, we combined the preprocessed comments and titles. This provided contextual information for each comment, helping the model understand the video associated with each comment to facilitate more effective comment analysis and topic identification.

### Topic Modeling Using BERTopic

To identify and group semantically related content, we used the Bidirectional Encoder Representations from Transformers for Topic Modeling (BERTopic) model, a topic modeling technique that integrates several machine learning components [37]. Specifically, we employed Uniform Manifold Approximation and Projection (UMAP) for dimensionality reduction, CountVectorizer for text tokenization, and Hierarchical Density-Based Spatial Clustering of Applications with Noise (HDBSCAN) for clustering [38, 39]. This method is particularly well-suited for analyzing short, informal texts such as YouTube comments, as it captures semantic relationships more effectively than traditional frequency-based models. The model generated topic clusters, each represented by a set of keywords that reflected dominant themes within the group. These keywords were then used to interpret and label the topics, contributing to our broader analysis of thematic patterns in the dataset [37]. Specifically, BERTopic with HDBSCAN as the clustering algorithm was used, which automatically determines the number of topics based on the structure of the data. Unlike K-means, which requires a predefined number of clusters, HDBSCAN offers greater flexibility but generates topic labels that are model-driven and not inherently consistent across different groups. As a result, the number of topics varies by group: 34 for English males, 52 for English females, 24 each for Korean males and females, 49 for the English younger generation (under 40, MZ generation), 33 for English old participants, and 24 each for the Korean MZ and Korean old groups. These variations reflect differences in discourse patterns and further explain why topic labels may not align directly across groups. For example, in total, the model identified 30 topics from the YouTube comments. Each topic was defined by a group of keywords that appeared frequently together. Perhaps, one topic included words like “lonely,” “divorce,” “middle age,” and “starting over,” suggesting a theme about emotional struggles and life changes. These keyword groups helped us understand what people were talking about and made it easier to label and interpret the main ideas in the comments.

**FIGURE 1:**
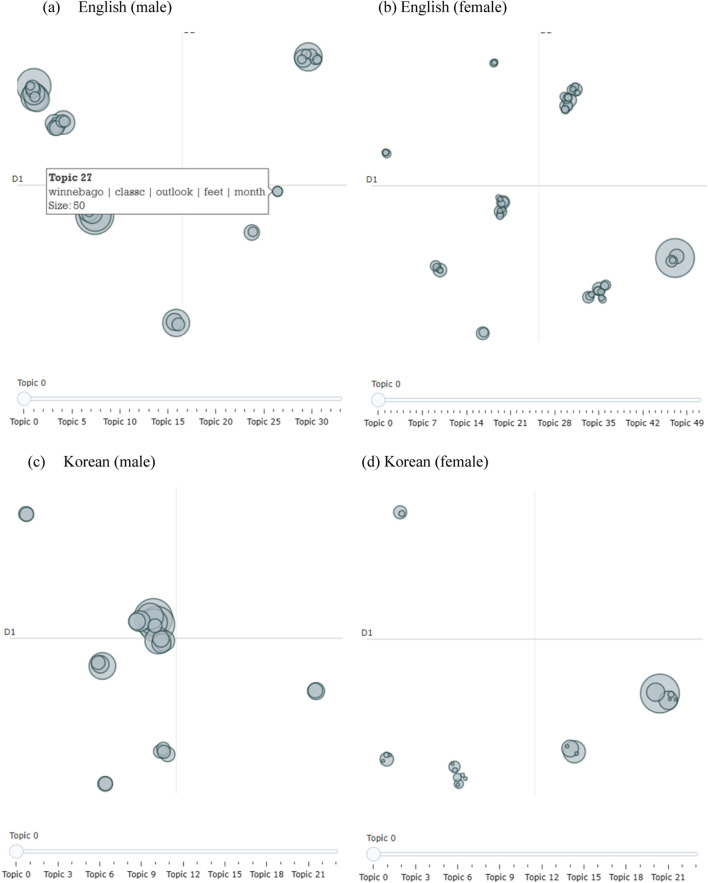
Visualization–intertopic distance map by sex. patterns of living alone in South Korea compared to other countries: a public health perspective and youtube topic modeling analysis, South Korea, 2025. **(A)** English (male). **(B)** English (female). **(C)** Korean (male). **(D)** Korean (female).

**FIGURE 2:**
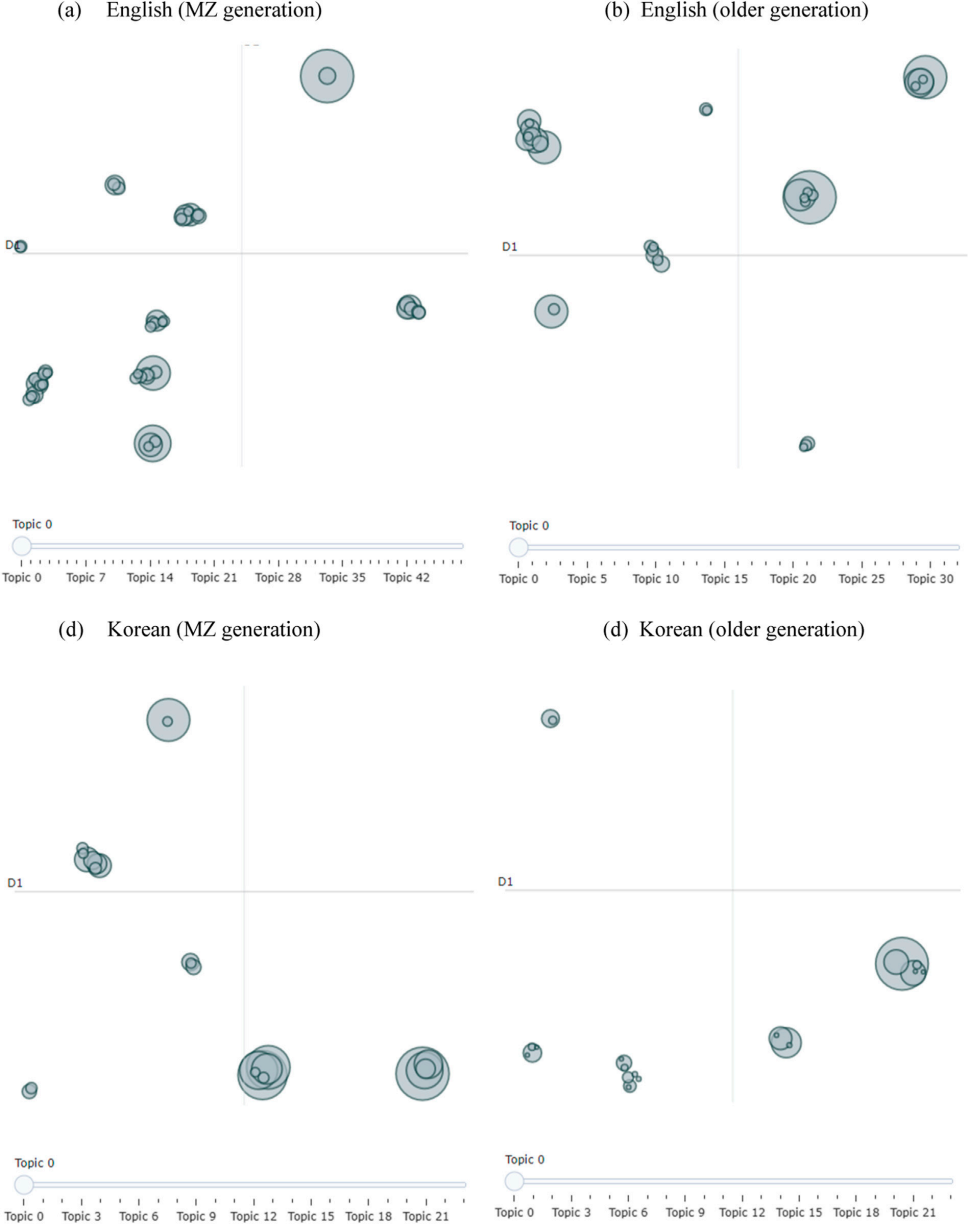
Visualization–intertopic distance map by generation. patterns of living alone in South Korea compared to other countries: a public health perspective and youtube topic modeling analysis, South Korea, 2025. **(A)** English (MZ generation). **(B)** English (older generation). **(C)** Korean (MZ generation). **(D)** Korean (older generation).

The inter-topic distance map, visual_topics, and visual_bar chart methods were used to visualize different topics between sex and generation. Figures 1, 2 illustrate the topics and keywords using the intertopic distance map. Each circle on the map represents a distinct topic, with the topic label or summary indicating the main theme of that topic. Larger circles indicate topics that appear more frequently in the data. The distance between circles reflects how similar or dissimilar the topics are; topics positioned closer together share more overlapping content, while those farther apart are more thematically distinct. We compared the responses between different generations. Although we aimed to include comments from individuals in their 30s, 40s, 50s, and older, we obtained a significant number of responses from those under 40 years of age. Consequently, we focused our comparison on two groups: older (40 years and above) and younger (under 40 years) generations. For instance, the results shown in Figures 1, 2 are consistent with those in Table 1.

**TABLE 1:** Topics categorized by sex and generation. patterns of living alone in South Korea compared to other countries: a public health perspective and youtube topic modeling analysis, South Korea, 2025.

Categories			Korean		English	
			Topics	Themes	Topics	Themes
Sex	male	Rank 1	Divorce, unemployment, old bachelor, daily life, work	Male Solitary Life and Employment Struggles	Car, spend, money, living, don, need, live, things, im, van	Van Life and Minimalist Living
		Rank 2	Divorce, old bachelor, life, readiness, vacation	Late-Life Singleness and Planning for the Future	Man, men, video, thing, videos, bro, nofap, im, life, just	Masculinity and Digital Brotherhood Culture
		Rank 3	unemployment, old bachelor, credit, truth, no jobs	Financial Hardship and Job Insecurity	Jayco, swift, class, motorhome, month, new, van, price, pay, buy	RV Lifestyle and Alternative Housing
		Rank 4	Lodging, travel, rent, question, solution	Housing Instability and Lifestyle Solutions	Women, men, woman, attractive, chasing, stop, want, starts, caring, dont	Dating Dynamics and Gender Relations
		Rank 5	Lodging, travel, rent, question, solution	Solo Travel and Living Arrangements	Beer, thirty, youtuber, chat, home, patio, fireside, rv, good, sanity	Online Leisure and Coping Through Content
	female	Rank 1	Old maid, single, alone, weekend, marriage	Female Singleness and Social Expectations	Accounting, public, external, work, career, industry, big, job, cpa, im	Professional Identity and Career Focus
		Rank 2	Old maid, single, mukbang (eatcasting), daily life, alone	Eating Alone and Emotional Solitude	Exam, cpa, pass, working, studying, fulltime, efficient, big, planning, tips	Test Prep and Career Advancement Strategies
		Rank 3	Simple, mukbang (eatcasting), daily life, saving, living alone	Frugal Lifestyle and Simple Living	Tutorial, smokey, eyes, makeup, circle, pink, mac, lenses, sexy, kardashian	Beauty Culture and Online Tutorials
		Rank 4	Seoul, old maid, single, narration, daily life	Urban Life and Female Solitude in Later Life	Ramadan, daily, month, welcome, amir, sara, fancy, youtuber, goals, keeping	Spirituality and Personal Development Online
		Rank 5	Mukbang (eatcasting), dinner, single, old, maid, saving	Food-Centered Routine and Economic Concerns	Eat, healthy, weight, day, food, recipes, lose, selfie, quarantine, youtuber	Health, Dieting and Self-Care
Generation	MZ	Rank 1	Saving, camping, tent, mukbang (eatcasting), vlog, appearance	Outdoor Leisure and Self-Sufficiency	Accounting, public, career, external, audit, industry, cpa, accounting, auditors, big	Career in Finance and Public Accounting
		Rank 2	Saving, mukbang (eatcasting), seeing, vlog, appearance	Frugality and Self-Image in Daily Life	Women, men, woman, chasing, stop, attractive, starts, text, caring, wrong	Relationship Pursuits and Frustrations
		Rank 3	Camping, sleeping in car, mukbang (eatcasting), Soju (a type of alcohol), solo	Solo Drinking and Mobile Living	Man, men, bro, video, nofap, greatest, thing, power, life, change	Male Empowerment and Discipline Discourse
		Rank 4	Old maid, 30s, old bachelor, drinking alone, single	Aging Alone and Unmarried Social Identity	Exam, cpa, pass, working, fulltime, studying, big, tips, efficient, planning	Studying Habits and Productivity Pressure
		Rank 5	Old maid, drinking alone, boss, unemployment, vlog	Social Isolation, Work, and Aging Stigma	Eemen, retention, sex, nofap, exposes, benefits, hurts, women, modern, energy	Sexual Self-Regulation and Motivation
	Old	Rank 1	Old maid in her 40s, single, vlog, unmarried, drinking alone, daily life, good life alone	Empowered Solitude in Middle Age	Texas, mexico, new, driving, state, park, youtuber, drive, trikeman, beautiful	Travel Vlogs and Scenic Exploration
		Rank 2	Unemployed, old bachelor’s daily vlog, unmarried life in his 40s, old maid, Seoul season	Male Aging, Job Loss, and City Living	Car, spend, don, money, living, need, things, live, im, dont	Financial Management in Minimal Living
		Rank 3	Married, unmarried, alone, not in their 40s, daily life, reasons, friends, vlog	Comparing Married and Single Life	Swift, jayco, motorhome, class, month, new, van, pay, years, price	Mobile Homes and Economic Trade-Offs
		Rank 4	Vlog, old bachelor in his 40s, daily life, divorce life, old maid, unmarried, unemployed person furniture	Daily Reality of Older Singles	Beer, thirty, youtuber, chat, fireside, sanity, session, rv, home, good	Recreational Solitude and YouTube Community
		Rank 5	Food, cooking, living, vlog, daily life, savings, simple diet, health	Frugal Diet and Health Maintenance	Youtuber, youtube, rv, videos, ing, channel, video, just, time, comment	Content Creation and Van Life Documentation

The largest circle corresponded to Rank 1 in the table for each category. For example, in the case of English females, Rank 1 (Accounting, public, CPA, work, big) has the highest frequency. Additionally, Rank 2 (Accounting, public, CPA, work, big) and Rank 4 (makeup, soft, abdul, glam, chatty) are positioned closely together, indicating a strong thematic relationship. In contrast, Rank 2 (home, new, decor, Ramadan, house) is located farther from Rank 1, reflecting greater thematic dissimilarity. The figures display the full set of topics identified for each group, numbered from topic 0 (the default output from BERTopic), whereas Table 1 presents only the top five most prominent topics for each group, presented in rank order. Specifically, we used BERTopic with HDBSCAN as the clustering algorithm, which automatically determines the number of topics based on the structure of the data. Unlike K-means, which requires a predefined number of clusters, HDBSCAN offers greater flexibility but generates topic labels that are model-driven and not inherently consistent across different groups. As a result, the number of topics varies by group: 34 for English males, 52 for English females, 24 each for Korean males and females, 49 for the English younger generation (under 40, MZ generation), 33 for English old participants, and 24 each for the Korean MZ and Korean old groups. These variations reflect differences in discourse patterns and further explain why topic labels may not align directly across groups.

## Results

A total of 2,736 videos accompanied by approximately 780,000 comments from Korea and 11,059 videos with 700,953 comments from English-speaking countries were included in the analysis. Fifteen topics were identified for each of the two groups of single-person households [1]: those observed among Korean speakers and [2] those observed among English speakers. Additionally, the topics were analyzed comparatively by sex and generation, distinguishing between older (40 years and above) and younger (under 40 years old) individuals. Topics were identified for eight groups, with the number of topics varying by group: 34 for English males, 52 for English females, 24 each for Korean males and females, 49 for the English younger generation (under 40, MZ generation), 33 for older English participants, and 24 each for the Korean MZ and older Korean groups. Table 1 presents the top five most prominent topic ranks identified within sex and generation categories for both the Korean and English-speaking groups. In Korea, topics related to the emotional and social dimensions of living alone were prevalent across both male and female respondents. Among Korean men, frequently occurring terms included “divorce,” “bachelor,” and other expressions reflecting single status and marital history. Korean women, in contrast, were more often associated with emotionally charged and identity-related terms such as “single,” “old maid,” “marriage,” “living alone,” and “alone.” The recurrence of such language suggests a gendered discourse surrounding social expectations, stigma, and emotional experiences of solitary living in the Korean cultural context.

In the English-speaking group, a different pattern emerged. Women engaging in English-language discussions often centered their comments around professional identity and work-related aspirations. Prominent keywords included “work,” “career,” “working,” and “CPA (Certified Public Accountant),” indicating a strong association between single living and professional development. For instance, revealed a high level of interest in facial cosmetics among English-speaking women, pointing to themes of self-presentation and consumer culture. Meanwhile, English-speaking men and women alike frequently discussed careers and professional growth, suggesting a broader cultural narrative linking independence with ambition and personal advancement.

These findings highlight meaningful differences in the thematic framing of single living, shaped by both cultural context (Korean vs. English-speaking environments) and demographic factors such as sex and generation. The results underscore how language and cultural background mediate the social construction of single-person households in online discourse.

Men commenting in both the Korean and English-language contexts frequently addressed practical and material concerns. Topics such as employment, income, and financial security were common, with keywords including “job,” “employment,” and “money” recurring across multiple topics. In addition to financial matters, male commenters also showed notable interest in leisure and transportation, discussing themes related to travel and vehicles; terms such as “lodging,” “travel,” “vans,” and “cars” appeared prominently. This suggests a pragmatic orientation toward independence and lifestyle interests among male participants, transcending linguistic or cultural boundaries.

In contrast, women in both language groups were more engaged with topics centered on food and eating. In the Korean-language data, the popular online phenomenon of “mukbang” (eating broadcasts) was mentioned in two separate topics, highlighting its cultural resonance and entertainment value. English-speaking women, meanwhile, frequently used words such as “food,” “eat,” and “recipes,” suggesting a parallel but culturally distinct engagement with food-related content. This emphasis on eating may reflect a mix of social, emotional, and lifestyle considerations that shape women’s online narratives about solo living.

Health-related topics revealed even more nuanced differences across groups. Among Korean males, conversations often touched on emotional and physical wellbeing, highlighting themes such as “happiness” and “health,” emotional expression through “tears,” and the practice of “eating small.” These discussions suggest an internal effort to manage wellbeing, particularly within the context of solitary living. For Korean women, health-related topics were more varied and emotionally layered. They frequently mentioned being “alone,” the pursuit of “happiness,” and their identity as “solo” individuals. Discussions of “health and mind” and experiences of “failure” further reflected a deeply introspective emotional landscape shaped by societal expectations. English-speaking men also engaged with health and lifestyle topics, though their concerns were often framed around self-discipline, religious values, and existential challenges. Their conversations included references to “sleeping” and “rest,” as well as “sex” and “retention,” reflecting an interest in self-control and personal development. Faith-based coping strategies appeared through mentions of “God,” “Christianity,” and “overcoming challenges,” while concerns about “poverty” and “security” pointed to a mix of personal growth and social vulnerability in their discourse.

English-speaking women expressed a strong focus on health-related concerns tied to personal wellbeing and identity. Their discussions frequently emphasized diet and wellness, with many references to eating habits, healthy living, weight management, and food. In addition, they showed a unique interest in genetic identity, mentioning terms such as “DNA,” “ancestry,” and “results,” which may reflect curiosity about self-discovery or health tracking. Emotional nuances emerged as well, with references to “shade” and “failure,” possibly pointing to underlying societal pressures or personal struggles.

Generational differences added further complexity to the topic landscape. In Korea, younger individuals were particularly drawn to leisure and contemporary solo-living activities, such as watching mukbang, engaging in solo drinking (referred to as *honsul*), camping, and discussions about saving money, indicating a shift toward a more autonomous and lifestyle-oriented discourse. However, generational norms and cultural values still appeared deeply embedded. Notably, both younger and older Korean generations frequently mentioned the term “old maid,” reflecting persistent societal stigma around unmarried women. The older generation was especially vocal about themes of “unemployment” and “marriage,” with these terms appearing consistently across multiple topics. This generational focus on economic instability and social roles likely mirrors broader societal anxieties tied to aging and marital status in the Korean context.

Regarding health-related topics, distinct patterns emerge across different groups. For Korean males, the themes of happiness and health, tears, and eating small amounts are notably present. In contrast, Korean females more frequently engage with topics such as alone, happiness, solo health and mind, and failure. Among English-speaking males, key health-related discussions center around sleeping and rest, sex and retention, single, god, Christianity, and overcoming challenges, as well as poverty and security. For English-speaking females, the most prominent topics include eating, healthy living, weight, food, DNA ancestry and results, shade, and failure.

## Discussion

This study analyzed the life interests and concerns of individuals living in single-person households in Korea and other countries, focusing on YouTube comments in English and Korean. Our findings revealed significant cultural differences in how living alone is discussed. In Korea, both men and women frequently associated living alone with marital status, with men using terms such as “divorce” and “bachelor,” whereas women referred to being “single,” “old maid,” and “marriage,” reflecting societal pressure around marital expectations and the stigma associated with women living alone [24]. By contrast, English-language comments from the US and UK more emphasized career and professional topics such as “work,” “career,” and “CPA,” with women also discussing beauty products such as makeup [26, 40]. These differences highlight how cultural values shape perceptions of living alone, with Korean culture focusing on societal pressures tied to marriage and family, whereas Western cultures emphasize individualism and career success [41, 42].

Topics by sex differences further illustrate how societal norms shape these experiences. Korean women’s frequent use of “old maid” highlights the pressure they face to marry, whereas Western women’s focus on career development reflects different social values ^22^. Despite these differences, shared concerns were observed across both cultures, including practical topics such as employment and financial stability, as well as shared interests in travel and vehicles such as “lodging,” “travel,” “vans,” and “cars” [41, 42]. Food also emerged as a common topic, although discussed in culturally distinct ways. In Korea, “mukbang” reflects a desire to recreate communal dining experiences in a digital setting, fostering parasocial connections and alleviating feelings of isolation [43]. By contrast, English-speaking users focused on “recipes” and “cooking,” emphasizing self-sufficiency and creativity in meal preparation [41, 42]. These differences suggest that while food is a shared interest, its social meaning varies significantly across cultures.

Furthermore, generational differences revealed variations in priorities. In Korea, younger individuals expressed an interest in leisure activities such as mukbang, solo drinking (honsul), and camping, reflecting lifestyle trends focused on self-enjoyment and socialization [44]. By contrast, middle-aged Koreans frequently used terms such as “old maid” and “unemployed,” signaling concerns over marriage and financial security later in life. These expressions suggest that solo living is perceived variously across age groups as either a chosen, empowering lifestyle or a condition associated with social and economic vulnerability. Such contrasts emphasize the importance of developing age-sensitive approaches in content, interventions, and support systems. While younger individuals may benefit from platforms that foster connection, personal growth, and flexibility, middle-aged adults may require targeted resources related to stability, healthcare, and long-term planning. Recognizing these life-stage-specific needs is crucial for avoiding one-size-fits-all assumptions and for more effectively supporting the diverse experiences of people living alone [45–47].

Social isolation emerged as a central underlying theme across many of the topics identified in this study, particularly among individuals living alone. While not always explicitly mentioned, keywords such as “lonely,” “alone,” “old maid,” and references to “mukbang” or solo activities suggest a perceived or experienced lack of social connection. This aligns with existing research indicating that living alone can increase the risk of social isolation, which is closely linked to poorer mental and physical health outcomes [48–51]. Cultural context appears to mediate the experience of isolation. Korean comments, for instance, often expressed anxiety around deviating from traditional family structures, while English-speaking users focused more on self-sufficiency and individual choice. These findings suggest that while living alone is a global trend, the experience of social isolation is shaped by deeply rooted cultural beliefs and social norms. Addressing this issue through community-based interventions and tailored support systems is crucial to reducing its health burden.

From a public health perspective, these nuanced cultural and generational differences underscore the importance of developing culturally sensitive and age-appropriate support systems. In South Korea, where traditional family roles remain influential, individuals, particularly women, living alone may face compounded emotional and social challenges. Conversely, Western societies often provide more robust community support focused on career and personal development, including varied programs and services [6, 41, 52, 53].

Proactive strategies are essential for improving the quality of life of individuals living alone. Consistent access to social connection programs, mental health resources, and economic support tailored to the needs of single-person households is critical [6, 52, 53]. Promoting the use of health management tools, ensuring regular health checkups, and preparing for emergencies such as natural disasters can significantly enhance safety and wellbeing [6, 52, 53]. Community-based interventions, including regular check-ins, emergency alert systems, and organized social activities, can provide vital support to reduce loneliness and isolation.

While living alone poses a range of challenges, these can be mitigated through comprehensive community-based support systems [52, 53]. Addressing the mental health risks associated with social isolation, such as depression and anxiety, requires ensuring access to appropriate resources and support services [52, 53]. As earlier mentioned, social isolation is a major concern and a well-documented risk factor for mental health issues such as depression and anxiety, which can significantly affect individuals’ wellbeing and overall quality of life Addressing these risks requires ensuring equitable access to appropriate resources, including mental health counseling, peer support groups, and structured social programs [49, 50, 54–56]. Fostering community engagement and regular opportunities for social interaction can further help alleviate loneliness and promote psychological resilience. In addition to social and emotional challenges, individuals living alone often face increased difficulty in managing daily responsibilities, such as attending medical appointments and coordinating care [53]. This highlights the need for supportive and accessible health management systems tailored to their needs. Economic insecurity, including poverty, underemployment, or unstable income, also poses a significant threat to stability and wellbeing among older adults living alone and must be addressed through policy and community-level solutions [52]. Emergency preparedness, including disaster planning, is also a vital safeguard for this population [41]. By proactively addressing these challenges, individuals living alone can enhance their quality of life and reduce health risks over time.

### Limitations

This study had several limitations. First, although YouTube comments do not represent the entire population, they provide spontaneous and candid insights into the lived experiences of individuals living alone [24]. These online expressions offer valuable perspectives that are often difficult to capture through traditional research methods, particularly in understanding the social realities and health needs of socially isolated individuals [25]. Recognizing the limitations of online data, such as sample bias and questions of representativeness, strengthens the study’s implications for developing targeted and culturally appropriate public health strategies [24, 25].

Second, the authenticity and reliability of comments are uncertain. Users may post misleading information or use pseudonyms, and comments frequently lack sufficient context for full interpretation. Language and cultural differences further complicate analysis, especially if translations are imperfect. Additionally, demographic details such as age and sex are neither consistently available nor verifiable, meaning our interpretations rely primarily on thematic and linguistic cues rather than confirmed participant characteristics, which may limit generalizability and introduce bias.

Third, comments reflect the views of YouTube users and may not be representative of other platforms or offline behaviors. The dynamic nature of online content, with comments and videos being edited or deleted, also affects data consistency. We also acknowledge that some users may voluntarily share personal details (e.g., age, sex, etc.); however, such information is neither consistently available nor verifiable. Consequently, our interpretation of the comments relies on thematic and linguistic cues rather than confirmed demographic data, which may introduce bias or limit the generalizability of the findings. Additionally, analyzing the content of the videos themselves could enhance the depth and accuracy of the analysis. While the current study is limited to user comments and video titles/descriptions due to data accessibility constraints, incorporating the actual video content could offer richer contextual information and a better understanding of how topics are framed, thereby improving the interpretation of the comment-based topic modeling results. Future research could address these limitations by conducting surveys, interviews, and focus groups to explore experiences of living alone across factors such as sex, age, and culture. Longitudinal studies could track changes in themes and perceptions over time, while comparative studies could clarify cultural influences. Finally, future research should focus on specific health issues and interventions for individuals living alone.

### Conclusions

This study explored patterns in the life interests and concerns of individuals living alone in Korea by comparing them with those observed in other cultures through English-language YouTube comments. Our findings revealed that discussions on living alone in Korea often emphasize personal and emotional experiences, whereas those in English-speaking countries tend to focus on professional achievements and physical appearance. Korean discussions frequently highlight terms related to social isolation, such as “single,” “old maid,” and “alone,” reflecting the strong cultural emphasis on family and social connections. By contrast, discussions in English-speaking countries prioritize career development and personal interests, reflecting a greater acceptance of diverse lifestyles and less stigma associated with living alone.

This study also highlighted that traditional Korean norms lead to more frequent discussions about the emotional challenges of being single, whereas Western countries offer more robust community support systems that promote professional and personal development. Addressing the challenges of living alone requires a multifaceted approach that includes improving social support networks, mental health resources, and emergency preparedness. Future research should explore these issues by examining various cultural contexts and investigating specific interventions to enhance the quality of life of individuals living alone.
